# Stereotactic body radiotherapy for stage I non-small-cell lung cancer using higher doses for larger tumors: results of the second study

**DOI:** 10.1186/s13014-017-0888-7

**Published:** 2017-09-11

**Authors:** Akifumi Miyakawa, Yuta Shibamoto, Fumiya Baba, Yoshihiko Manabe, Taro Murai, Chikao Sugie, Takeshi Yanagi, Taiki Takaoka

**Affiliations:** 1 Department of Radiology, Nagoya City University Graduate School of Medical Sciences, 1 Kawasumi, Mizuho-cho, Mizuho-ku, Nagoya, 467-8601 Japan; 2 Department of Radiology, Nagoya City West Medical Center, 1-1-1, Hirate-cho, Kita-ku, Nagoya, 462-8508 Japan

**Keywords:** Non-small-cell lung cancer, Stereotactic body radiotherapy, Dose escalation, Overall survival, Toxicity

## Abstract

**Background:**

Efficacy of stereotactic body radiotherapy (SBRT) in stage I non–small-cell lung cancer (NSCLC) has almost been established. In Japan, the protocol of 48 Gy in 4 fractions over 4 days has been most often employed, but higher doses may be necessary to control large tumors. Previously, we conducted a clinical study using SBRT for stage I NSCLC employing different doses depending on tumor diameter, which was closed in 2008. Thereafter, a new study employing higher doses has been conducted, which is reported here. The purpose of this study was to review the safety and effectiveness of the higher doses.

**Methods:**

We escalated the total dose for the improvement of local control for large tumors. In this study, 71 patients underwent SBRT between December 2008 and April 2014. Isocenter doses of 48, 50, and 52 Gy were administered for tumors with a longest diameter of < 1.5 cm, 1.5–3 cm, and > 3 cm, respectively. It was recommended to cover 95% of the PTV with at least 90% of the isocenter dose, and in all but one cases, 95% of the PTV received at least 80% of the prescribed dose. Treatments were delivered in 4 fractions, giving 2 fractions per week. SBRT was performed with 6-MV photons using 4 non-coplanar and 3 coplanar beams.

**Results:**

The median follow-up period was 44 months for all patients and 61 months for living patients. Overall survival (OS) was 65%, progression-free survival (PFS) was 55%, and cumulative incidence of local recurrence (LR) was 15% at 5 years. The 5-year OS was 69% for 57 stage IA patients and 53% for 14 stage IB patients (*p* = 0.44). The 5-year PFS was 55 and 54%, respectively (*p* = 0.98). The 5-year cumulative incidence of LR was 11 and 31%, respectively (*p* = 0.09). The cumulative incidence of Grade ≥ 2 radiation pneumonitis was 25%.

**Conclusions:**

Our newer SBRT study yielded reasonable local control and overall survival and acceptable toxicity, but escalating the total dose did not lead to improved outcomes.

**Trial registration:**

UMIN000027231, registered on 3 May 2017. Retrospectively registered.

## Background

Stereotactic body radiotherapy (SBRT) is used for the treatment of primary cancer and oligometastatic disease [1, 2]. In particular, the efficacy of SBRT in stage I non-small cell lung cancer (NSCLC) has been generally established [3–12]. In Japan, the protocol of 48 Gy in 4 fractions over 4 days is the most widely employed for both stage IA and IB NSCLC [3, 7, 10]. However, it was found that the outcomes of stage IB patients were worse than those of stage IA patients at the same dose [4, 7]. Therefore, we postulated that higher doses may be necessary to control larger tumors.

We evaluated the clinical outcomes of SBRT for stage I NSCLC employing different doses depending on tumor diameter in our first study [13, 14]. Based on these results, we escalated the total dose in order to improve outcomes in a second study. In addition, we changed the dose calculation algorithm from pencil beam convolution to the analytical anisotropic algorithm (AAA), which is expected to improve dose distribution. We hypothesized that dose escalation using the AAA algorithm would be feasible and lead to improved outcome. Eight years have passed since we started the second protocol, so we analyzed the clinical outcomes of patients involved in the second study.

## Methods

### Study design and eligibility criteria

The study protocols were approved by the institutional review board (NCU-0803). Informed consent was obtained from all patients before SBRT. The present second study aimed to accrue 180 patients in accordance with the first study [14, 15]; however, because many affiliated hospitals started SBRT for lung cancer during the last 10 years, patient accrual became much slower at our institution. So, we decided to perform an interim analysis for the second study, the results of which are reported here, in order to evaluate the adequacy of continuing the protocol.

Eligibility criteria were similar to the previous study: (1) histologically confirmed stage I NSCLC diagnosed according to the 7th TNM classification of lung cancer by the Union for International Cancer Control by chest and upper abdomen CT, brain MRI, and bone scintigraphy or FDG-PET; (2) greatest tumor diameter ≤ 5 cm; (3) World Health Organization performance status (PS) ≤ 2 or PS 3 when its cause was not a pulmonary disease; (4) no prior therapy and no concurrent malignancy; and (5) arterial oxygen pressure ≥ 60 mmHg, and forced expiratory volume in 1 s ≥ 700 ml.

### Patient characteristics

Seventy one patients underwent SBRT between December 2008 and April 2014. All patients completed the planned treatment. Patients were deemed medically inoperable when they had a poor pulmonary function (ratio of forced expiratory volume in 1 s to forced vital capacity < 60% and/or percent vital capacity < 75%) or other debilitating conditions that precluded surgery. The patient and tumor characteristics are shown in Table 1. The tumor location was classified into central or peripheral in accordance with Radiation Therapy Oncology Group criteria [16].

**Table 1:** Patients and tumor characteristics

Characteristics	
Patient number	71
Age (years), range (median)	55–89 (77)
Gender, male/female	51/20
Performance status (0/1/2)	32/33/6
T stage (T1a/T1b/T2a)	24/33/14
Total dose (48 Gy/50 Gy/52 Gy)	6/51/14
Tumor location	
Center/periphery	8/63
Operability	
Operable/inoperable	24/47
Operable (T1a/T1b/T2a)	12/8/4
Inoperable (T1a/T1b/T2a)	12/25/10
Histology	
Adeno/squamous/NSCLC	50/14/7

*Adeno* adenocarcinoma, *squamous* squamous cell carcinoma, *NSCLC* unclassified non-small-cell lung cancer

### Treatment

Our treatment methods were described in detail previously [13, 15]. We used the BodyFIX system (Medical Intelligence, Schwabmuenchen, Germany) for patient immobilization. The visible gross tumor volume on CT during three phases (normal breathing and breath holding during the expiratory and inspiratory phases) was superimposed to represent the internal target volume (ITV). Breath-holding-phase CT images were used to ensure the range of tumor motion. During the first study, we had confirmed that a forced inspiration/expiration breath hold would not overestimate the tumor motion and therewith systematically overestimate the margins, by using fluoroscopy [17]. The planning target volume (PTV) margin for the ITV was 5 mm in the lateral and anteroposterior directions and 5–10 mm in the craniocaudal direction. Forward planning was performed using a 3-dimensional treatment planning system (Eclipse Version 7.5.14.3, Varian Medical Systems, Palo Alto, California, USA). Fixed 3 coplanar and 4 non-coplanar beams were used in all cases.

For verification of tumor positions, we used the simulator CT at the first and third treatments in addition to megavoltage portal imaging at every treatment throughout the study period. The patients underwent registration in the CT simulator room, and repositioning was performed whenever necessary. Then, they were carefully transferred to the linac room with a stretcher. SBRT was delivered by CLINAC 23EX (Varian Medical Systems, Palo Alto, California, USA) with 6-MV photon beams and it was delivered with 4 fractions. In principle, the respective fractions were delivered at intervals of ≥ 72 h to allow reoxygenation of hypoxic tumor cells [14], but owing to national holidays, patient schedule convenience, and machine availability, the actual overall treatment period was 8–20 days (median, 11 days). The total dose at the isocenter was increased to 48 Gy for tumors with a maximum diameter < 1.5 cm and 50 Gy for tumors of 1.5–3 cm. For those > 3 cm, the total dose remained at 52 Gy. The dose calculation algorithm was AAA.

It was recommended to cover 95% of the PTV with at least 90% of the isocenter dose, and, in all cases, 95% of the PTV received at least 80% of the prescribed dose. However, the dose was 79.2% in one case. Dose constraints for normal tissues were: (1) volume of the lung receiving 20 Gy, ≤ 20%; (2) 40 Gy for < 1 cm^3^ of the pulmonary artery and esophagus; (3) 36 Gy for < 10 cm^3^ of the stomach; and (4) maximum cord dose < 18 Gy, in accordance with the first study [13, 14].

### Evaluation

Chest and upper abdominal CT was performed at 2-month intervals until 6 months, and every 2–4 months thereafter. FDG-PET was performed whenever necessary. Local recurrence was diagnosed using serial CT examinations combined with FDG-PET and/or biopsy, as described in detail previously [18]. Pleuritis carcinomatosa unaccompanied by local recurrence was regarded as distant metastasis. Toxicity was evaluated using the Common Terminology Criteria for Adverse Events version 4. Follow-up after 5 years was conducted at the discretion of the attending radiation oncologist.

### Statistical analysis

Overall survival (OS) and progression-free survival (PFS) were calculated from the start of SBRT using the Kaplan–Meier method. The log-rank test was used to compare these curves. A Fine and Gray competing-risks regression model was used to estimate and compare cumulating incidences of local recurrence (LR), thereby considering patient death as a competing risk. Incidence of complications was compared using Fisher exact test. All statistical analyses were carried out using R version 2.13.0 for Windows (The R Foundation for Statistical Computing, Vienna, Austria). *p-*values of < 0.05 were defined as significant.

## Results

### Efficacy

**Fig. 1:**
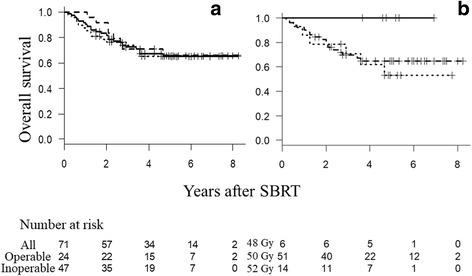
**a** Overall survival curves for all patients (*solid line*), operable patients (*dashed line*) and inoperable patients (*dotted line*). **b** Overall survival curves for patients treated with 48 Gy/4 Fr (*solid line*), 50 Gy/4 Fr (*dashed lin*e), and 52 Gy/4 Fr (*dotted line*)

**Fig. 2:**
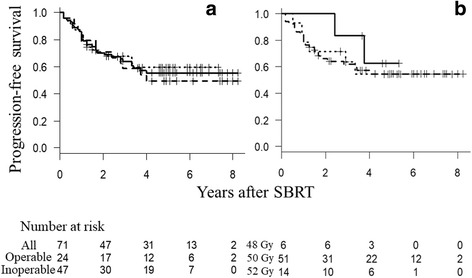
**a** Progression-free survival curves for all patients (*solid line*), operable patients (*dashed line*) and inoperable patients (*dotted line*). **b** Progression-free survival curves for patients treated with 48 Gy/4 Fr (*solid line*), 50 Gy/4 Fr (*dashed line*), and 52 Gy/4 Fr (*dotted line*)

**Fig. 3:**
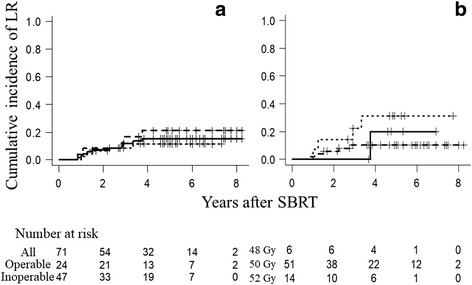
**a** Cumulative incidences of local recurrence (LR) for all patients (*solid line*), operable patients (*dashed line*) and inoperable patients (*dotted line*). **b** Cumulative incidences of local recurrence for patients treated with 48 Gy/4 Fr (*solid line*), 50 Gy/4 Fr (*dashed line*), and 52 Gy/4 Fr (*dotted line*)

The median follow-up period was 44 months for all patients and 61 months for living patients. At 5 years, OS was 65%, PFS was 55%, and the cumulative incidence of LR was 15%. Figs. 1a, 2a and 3a show OS, PFS, and cumulative incidence of LR in all patients, operable patients, and medically inoperable patients. The OS, PFS, and cumulative incidence of LR did not differ between the operable patients and inoperable patients (Table 2). Figs. 1b, 2b and 3b show OS, PFS, and cumulative incidence of LR in patients treated with 48, 50, and 52 Gy. The 3- and 5-year data in these patients are shown in Table 3. The OS, PFS, and cumulative incidence of LR did not differ among the 3 groups. The 5-year OS was 69% for 57 stage IA patients and 53% for 14 stage IB patients (*p* = 0.44). The 5-year PFS was 55 and 54%, respectively (*p* = 0.98). The 5-year cumulative incidence of LR was 11 and 31%, respectively (*p* = 0.09).

**Table 2:** Three and 5-year data in all, operable, and inoperable patients

	All patients (*n* = 71)	Operable patients (*n* = 24)	Inoperable patients (*n* = 47)	*p*-value^a^
OS (%) at 3/5 years	73/65	71/66	74/65	0.78
PFS (%) at 3/5 years	64/55	58/49	68/60	0.61
Cumulative incidence of LR (%) at 3/5 years	12/15	17/21	9/12	0.34

*OS* Overall survival, *PFS* Progression-free survival, *LR* Local recurrence

^a^Difference between operable and inoperable patients

**Table 3:** Three and 5-year data in patients receiving 48 Gy/4 Fr, 50 Gy/4 Fr, and 52 Gy/4 Fr

	48 Gy/4 Fr (*n* = 6)	50 Gy/4 Fr (*n* = 51)	52 Gy/4 Fr (*n* = 14)
OS (%) at 3/5 years	100/100	70/65	71/53
PFS (%) at 3/5 years	83/63	62/55	64/54
Cumulative incidence of LR (%) at 3/5 years	0/20	10/10	22/31

*OS* Overall survival, *PFS* Progression free survival, *LR* Local recurrence

### Toxicities

The cumulative incidence of Grade ≥ 3 radiation pneumonitis was 5.6% (4 of 71 patients; 3 Grade 3, and 1 Grade 5). The cumulative incidence of Grade ≥ 2 radiation pneumonitis was 25%. Other adverse events were as follows: Grade ≥ 2 pleural effusion was detected in 2 patients; Grade 1 atelectasis was 1 patients; Grade ≥ 2 dermatitis were observed in 4 patients; Grade 2 rib fractures were seen in12 patients; and Grade 1 soft tissue swelling was detected in 2 patients, respectively.

Regarding the differences due to tumor location, the incidence of Grade ≥ 2 radiation pneumonitis was 50% (4/8) and 22% (14/63; *p* = 0.09) for patients with a central tumor and those with a peripheral tumor, respectively. The incidence of Grade ≥ 3 radiation pneumonitis was 13 and 4.7%, respectively (*p* = 0.37). Other adverse events for central versus peripheral tumors were as follows: Grade ≥ 2 pleural effusion, 0 versus 2 (*p* = 0.61); Grade 1 atelectasis, 0 versus 1 (*p* = 0.71); Grade ≥ 2 dermatitis, 1 versus 3 (*p* = 0.37); Grade 2 rib fractures, 3 versus 9 (*p* = 0.10); and Grade 1 soft tissue swelling, 0 versus 2 (*p* = 0.61).

## Discussion

In view of the poorer outcomes of stage IB NSCLC patients compared to those of stage IA patients, we used protocols to employ higher doses for larger tumors. Based on the toxicity results of the first study, we considered that the doses for T1 tumors could be further increased, and the total dose was escalated from 44 to 48 Gy for tumors < 1.5 cm and from 48 to 50 Gy for 1.5–3 cm tumors [13, 15]. On the other hand, dose escalation was deferred for T2 tumors because Grade ≥ 2 radiation pneumonitis was observed in 30% of T2a patients in the first study [13]. For T2a tumors, we planned to combine SBRT with S-1 chemotherapy, but the protocol was not approved by the institutional review board, so we used the same 52 Gy dose in the second study as in the first study. Therefore, the possible advantages of the second protocol were dose escalation by 2 to 4 Gy and the use of AAA for T1a/b tumors and the latter alone for T2a tumors. Pencil beam algorithm tends to overestimate the dose distribution in PTV, and AAA can provide more accurate dose distributions [19, 20]. Using AAA could actually lead to higher dose delivery, and the isocenter dose is usually allowed to be considerably higher in order to deliver as low doses as possible to surrounding structures [21]. On the other hand, our isocenter dose prescription method that recommended to cover 95% of the PTV by at least 90% of the isocenter dose might lead to unnecessary dose spread to the surrounding tissues [22].

Although we attempted at dose escalation in the present study, other groups are using still higher doses. In a recent Japanese Clinical Oncology Group study 0702, dose escalation was attempted to determine the recommended dose for T2N0M0 patients [23]. The continual reassessment method was used, and the recommended dose was determined to be 55 Gy in 4 fractions as a dose covering 95% of the PTV for tumors < 100 cm^3^. The maximum and the isocenter doses were both 66.8 Gy in a patient with a prescribed dose of 55 Gy. In other studies from Western countries, 54 or 60 Gy in 3 fractions as the dose covering 95% of the PTV or to the 80% isodose line was used for peripheral tumors, while slightly lower doses were prescribed for centrally-located tumors, T2 tumors, and tumors with chest wall invasion; all of them reported acceptable toxicities and favorable outcomes [6, 9, 24]. Since we prescribed the doses to the isocenter, the doses covering 95% of the PTV and doses to the 80% isodose line were still lower; our doses covering 95% of the PTV were 89.7% ± 2.8% (mean ± standard deviation) of the isocenter dose, and the doses to the 80% isodose line were about 80% of the isocenter dose. Therefore, further dose escalation should be considered in future investigations.

In attempting at dose escalation, the relatively high incidence of Grade ≥ 2 radiation pneumonitis may be a problem. However, one reason for the high incidence may be that all radiation pneumonitis events to which corticosteroids were prescribed by attending physicians were regarded as Grade 2 toxicity; in some cases, steroids might have been unnecessary. Another reason may be that our method to control respiratory tumor motion was not sufficient, since we only used abdominal compression and shallow breathing. Hence, the ITV margins became larger. In more recent patients not included in this analysis, we have used a breath-holding method, so the ITV margins have become smaller. Nevertheless, in general, complication rates in the present study were not greatly different from those of the first study [13, 15]. Grade ≥ 2 radiation pneumonitis and rib fractures in patients with a tumor adjacent to the chest wall can develop at certain rates, so it may be difficult to decrease them. The incidence of Grade ≥ 2 rib fracture was indeed higher in the present study [13, 15]. On the other hand, skin, and esophageal toxicities may be avoided by taking care not to produce hot spots. Although there were no differences in toxicity between centrally located and peripheral tumors in this study, several studies suggest that SBRT for a central lesion increases a risk for severe radiation injury of normal tissues, such as the lung, large airways, great vessels, esophagus and heart [16, 24, 25]. So, larger fraction numbers may generally be recommended for central tumors.

To evaluate efficacy of the newer protocol, we reanalyzed 113 patients enrolled in our first study at our own institution thereby updating the follow-up data. In the first study, the median follow-up period was 51 months for all patients and 73.5 months for living patients. At 5 years, OS was 53% in the first study, while it was 65% in the present study (*p* = 0.18). PFS was 44% vs 55% (*p* = 0.09), and the cumulative incidence of LR was 15% in both studies (*p* = 0.82). Although OS and PFS at 5 years and later tended to be higher in the second than in the first study, there were no differences. The curves for the second study tended to lie above (data not shown), and the lack of significance may be due to the patient number. If more patients are included in the second study, the differences may become significant. In this study, the OS, PFS, and cumulative incidence of LR did not differ between the operable and inoperable patients. So, the beneficial effect of SBRT especially in inoperable patients seems remarkable. Conventional radiotherapy yielded 5-year survival rates of 30% or lower in stage I NSCLC patients [26], so the advent of SBRT is quite valuable for these patients.

OS, PFS, and LR rates were also not greatly different between stage IA and stage IB patients. However, the LR rate for stage IB tended to be higher. If more patients are included, the differences may become significant. Nevertheless, improvement in treatment outcome may be relatively small, if any, even if we continue to use this second protocol. Improvement in local control is desirable for T2a tumors, and to achieve this, further dose escalation should be attempted. The use of more fraction numbers or particle therapy may be recommended [27, 28], but even when these policies are adopted, dose escalation should be investigated [29].

There are several limitations for this study. First, the sample size was relatively small; many neighboring hospitals started SBRT, and patient accrual slowed down. Second, low doses were used compared to other recent studies in the literature. Third, the high rate for Grade *≥* 2 pneumonitis may not reflect the actual rate due to the early administration of steroids. Controlling the steroid administration may be necessary in future clinical studies.

## Conclusion

The present study yielded favorable outcomes. However, escalating the total dose did not lead to improved outcomes, although OS and PFS at 5 years and later tended to be higher in the present than in the first study. Further dose escalation should be investigated in future studies.

## Abbreviations

AAA: Analytical anisotropic algorithm
CT: Computed tomography
FDG-PET: ^18^F–fluoro-deoxyglucose-positron emission tomography
Gy: Gray
ITV: Internal target volume
LR: Local recurrence
MV: Mega voltage
NSCLC: Non-small cell lung cancer
OS: Overall survival
PFS: Progression-free survival
PS: Performance status
PTV: Planning target volume
SBRT: Stereotactic body radiotherapy
